# FIGO statement on respectful care: Addressing disrespectful maternity care

**DOI:** 10.1002/ijgo.70513

**Published:** 2025-09-30

**Authors:** Jezid Miranda, Hasmik Bareghamyan, Michelle N. S. Therrien, Andre Lalonde, Margit Steinholt, Francesca Palestra, Debra Pascali‐Bonaro, Mindaugas Kliucinskas, María A. Basavilvazo Rodríguez, Garang Ajak, Eliana Amaral, Pius Okong, Birgitta Essèn, Bo Jacobsson, Suellen Miller, the FIGO Committee on Health Systems Strengthening and Respectful Care

**Affiliations:** ^1^ Department of Obstetrics and Gynecology, Grupo de Investigación Prenatal Universidad de Cartagena Cartagena de Indias Colombia; ^2^ Department of Obstetrics and Gynecology Serena del Mar Medical Center and the Santa Fe Foundation of Bogota Bogotá Colombia; ^3^ Department of Obstetrics and Gynecology N1 Yerevan State Medical University Yerevan Armenia; ^4^ Safe Matherhood Program University of California San Francisco San Francisco California USA; ^5^ International Federation of Gynecology & Obstetrics London UK; ^6^ Department of Obstetrics and Gynecology University of Ottawa Ottawa Ontario Canada; ^7^ Department of Obstetrics and Gynecology McGill University Montreal Quebec Canada; ^8^ Department of Obstetrics and Gynecology Helgeland Hospital Sandnessjoen Norway; ^9^ Department of Medicine and Health Sciences Norwegian University of Science and Technology Trondheim Norway; ^10^ Department of Global Public Health Karolinska Institutet Stockholm Sweden; ^11^ International MotherBaby Childbirth Organization USA; ^12^ Department of Obstetrics and Gynecology Lithuanian University of Health Sciences Kaunas Lithuania; ^13^ Department of Perinatology La Raza National Medical Center of Obstetrics and Gynecology Distrito Federal Ciudad de Mexico Mexico; ^14^ Department of Obstetrics and Gynecology University of Juba Yuba South Sudan; ^15^ Department of Obstetrics and Gynecology University of Campinas São Paulo Brazil; ^16^ Department of Obstetrics and Gynecology St Francis Hospital Nsambya Kampala City Uganda; ^17^ Department of Women's and Children's Health Uppsala University Uppsala Sweden; ^18^ Department of Obstetrics and Gynaecology Region Västra Götaland, Sahlgrenska University Hospital Gothenburg Sweden; ^19^ Department of Obstetrics and Gynecology, Institute of Clinical Sciences, Sahlgrenska Academy University of Gothenburg Gothenburg Sweden; ^20^ Department of Genetics and Bioinformatics, Division of Health Data and Digitalisation Institute of Public Health Oslo Norway

**Keywords:** birth, childbirth, evidence‐based treatment, obstetric violence, respectful maternity care

## Abstract

The International Federation of Gynecology and Obstetrics (FIGO) Committee on Health Systems Strengthening and Respectful Care recognizes the detrimental effects of disrespectful and abusive practices within maternity care on maternal and neonatal health outcomes. In response, the committee advocates for the implementation of strategic, evidence‐based interventions aimed at safeguarding women from substandard and disrespectful treatment during pregnancy, childbirth, and the postpartum period. This statement presents FIGO's recommendations for adopting respectful maternity care into health systems. The proposed policy interventions and clinical strategies are designed to foster compassionate, person‐centered, and culturally competent care, thereby contributing to improved maternal and perinatal outcomes globally.

## 1 RECOMMENDATIONS

FIGO promotes significant improvements in maternity and neonatal care through a series of strategic recommendations:

1. Adopt national standards to improve the quality of maternal and newborn care in healthcare facilities.
2. Implement and monitor the International Childbirth Initiative framework (12 steps for safe and respectful care) to improve care at the facility and community levels (Table 1).^1^, ^2^
3. Develop and implement local and national pre‐service and in‐service training programs to enhance the skills necessary to provide respectful, evidence‐based, trauma‐informed, and culturally appropriate care.
4. Healthcare professionals should ensure that women's decision‐making is centered; prenatal care providers orient women around the choices they have and prepare women for physiological labor and birth, as well as for decisions that might arise in emergency situations.
5. Ensure all women and their newborns can access free or affordable, high‐quality health services with respect and cost transparency throughout pregnancy, birth, postpartum, and neonatal care.
6. Integrate respectful care principles explicitly into neonatal care protocols, ensuring newborns receive not only clinically appropriate treatment but also compassionate, non‐discriminatory, and developmentally sensitive care from birth.

## 2 INTRODUCTION

While women might now benefit from improved access to evidence‐based prenatal, intrapartum, and postnatal care, which have the potential to reduce maternal and neonatal mortality, rates have stagnated. This is a concerning trend highlighted in recent reports by the World Health Organization (WHO).^3^, ^4^ It has been theorized that a lack of respectful, compassionate care that meets women's emotional and psychological needs during pregnancy and childbirth has contributed to this stagnation.^5^, ^6^ Respectful care is essential to the provision of comprehensive, quality maternal health care. Women are entitled to a positive childbirth experience; however, global reports persistently indicate that women experience physical, emotional, and psychological distress at unacceptable rates due to numerous factors that create a harmful environment.^7^, ^8^

A critical component of quality of care is the experience of care, which has a complex interaction with pregnancy outcomes. Experience of care includes effective communication, respect and preservation of dignity, and emotional support.^9^ Every pregnant woman and newborn should be offered evidence‐based clinical and non‐clinical practices delivered in a humane, respectful, and supportive environment, giving women, their families, and healthcare providers a positive experience of care.^6^, ^9^

Recognizing and upholding the rights of women, infants, and their families are essential for strengthening health systems and fostering societal progress.^10^ As a leading global professional organization, the International Federation of Gynecology and Obstetrics (FIGO) is committed to protecting women's right to a dignified and healthy pregnancy, birth, and postnatal experience. FIGO makes policy recommendations for member societies and healthcare providers, promoting knowledge exchange across societies and leading global efforts to transform obstetric care. This statement considers the necessary actions to foster an environment of respect and safety and safeguard the fundamental rights of the mother‐baby dyad, building on prior FIGO guidelines and statements.^1^, ^11^

## 3 TERMINOLOGY

Terminology to describe disrespectful care and mistreatment of women and newborns during pregnancy and childbirth, includes “disrespect and abuse,” “substandard care,” and “obstetric violence”.^5^, ^12^ Selecting terminology that is well received and understood by maternity healthcare providers is essential to maintain an open dialogue between providers, patients, and their families. The term “disrespectful care” encompasses intentional behavior and unintentional consequences, including omissions in care. “Substandard care” refers to care that does not meet recommendations for best practice. The term “obstetric violence” is used to refer to acts of physical or psychological abuse and includes treatment without meaningful consent by any healthcare providers (including nurses, midwives, doctors, and administrators) during the maternity cycle. However, the term obstetric violence is sometimes negatively perceived by healthcare providers, creating a barrier to addressing challenges and creating a compassionate environment for birth.^12^, ^13^ Healthcare providers have shown a better understanding and response to “disrespectful maternity care” compared to “obstetric violence,” and it has been accepted as preferred terminology by other professional associations.^12^, ^13^ We advocate for the term “disrespectful care” as it captures the spectrum of negative experiences women may endure, ranging from mild offenses to severe abuses. The Bowser and Hill categories of disrespect and abuse highlight the common occurrence of disrespectful incidents, aiding healthcare providers to recognize and address it with better comprehension.^5^

### 3.1 Disrespect in maternity care

Disrespect and abuse (D&A) in facility‐based maternity care have been classified into the following categories: discrimination, non‐consented care, physical abuse, non‐confidential care, non‐dignified care, abandonment, and detention (Box 1).^5^ National and international agencies have adopted this D&A definition and classification, including the WHO and the White Ribbon Alliance.^14^ These categories describe not only ethical–medical issues but also treatment that might be illegal under regional and national legal codes.^14^ While disrespectful care occurs throughout the healthcare system, it receives attention in maternity care due to the vulnerability of women during childbirth and its high prevalence around the world.

**TABLE 1:** 12 Steps to Safe and Respectful MotherBaby‐Family Maternity Care (summary).

**Step 1:** Treat women and newborns with compassion, respect, dignity; provide informed choice.
**Step 2:** Offer free or affordable care with cost transparency and without discrimination
**Step 3:** Provide MotherBaby‐Family centered maternity care.
**Step 4:** Offer continuous support
**Step 5:** Provide nonpharmacological comfort and pain relief measures, and pharmacological measures with appropriate monitoring if available.
**Step 6:** Provide care based on evidence‐based practice.
**Step 7:** Avoid practices that are potentially harmful or practices without evidence of benefit.
**Step 8:** Enhance wellness and prevent illness.
**Step 9:** Provide emergency maternal and neonatal care.
**Step 10:** Have a supportive human resource policy.
**Step 11:** Provide a continuum of collaborative care.
**Step 12:** Promote breastfeeding and skin‐to‐skin care, referring to the Baby Friendly Hospital initiative
*Please refer to the full‐length document for additional details (icichildbirth.org)*

BOX 1 **Categories of disrespect and abuse (Bowser and Hill^5^)**

-  Discrimination (based on social status, ethnicity, culture, or language)
-  Non‐consented care (limiting women's ability to make decisions; failing to provide adequate information)
-  Physical abuse (slapping, pinching, inappropriate physical contact, overmedicalized care, lack of pain management)
-  Non‐confidential care (overcrowding, failure to provide physical privacy with curtains and blankets)
-  Non‐dignified care (leaving women in physically exposed or vulnerable positions, failing to speak to women, speaking rudely or dismissively),
-  Abandonment (neglect, failing to respond to requests for help, denying women companions such as the father, partner, family member, and doula)
-  Detention (refusing to let women leave or withholding the newborn for lack of payment)^5^

### 3.2 Substandard care

**FIGURE 1:**
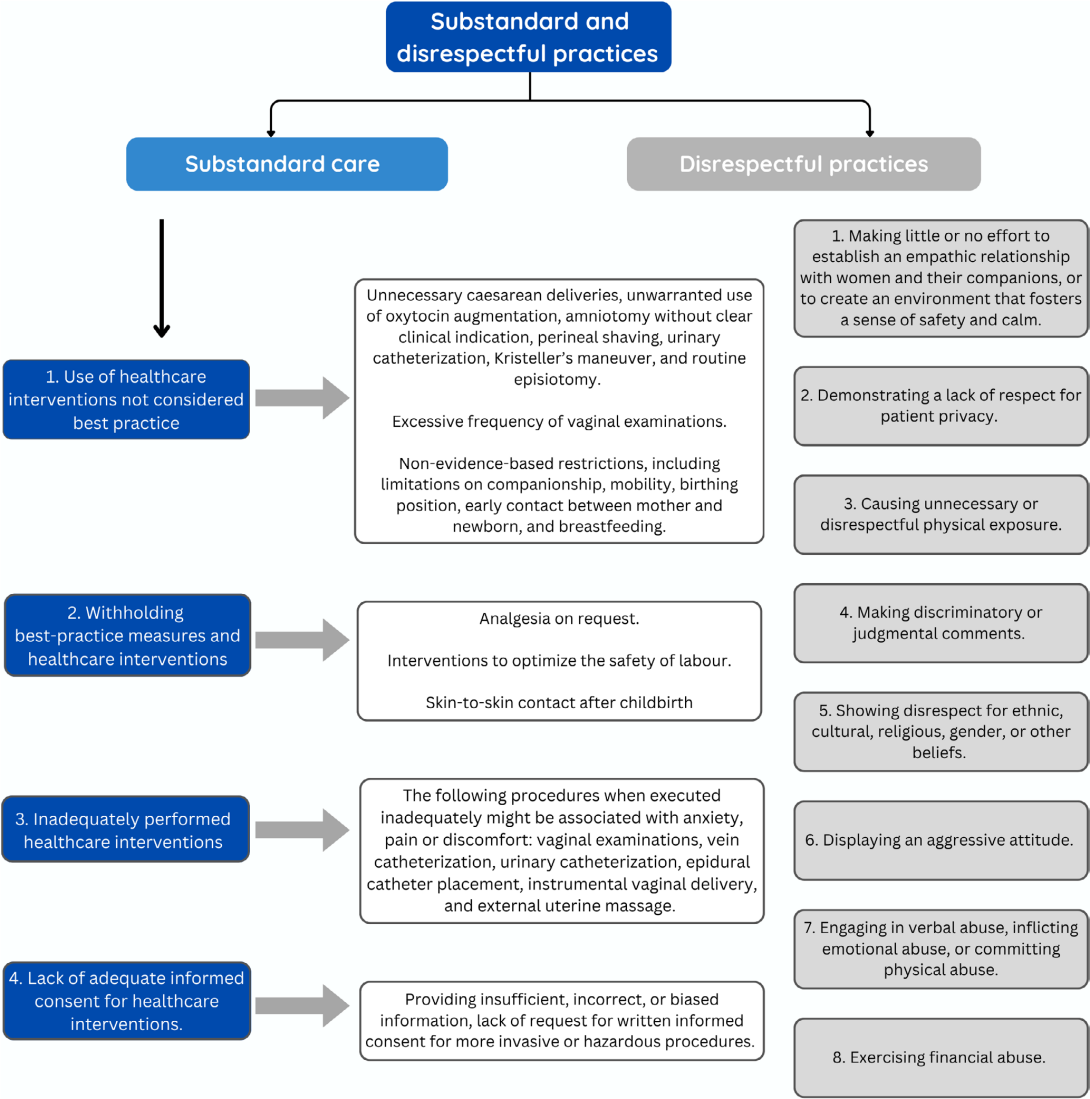
Substandard and disrespectful practices (adapted from Tables 1 and 2 [Ayres‐de‐Campos et al.^12^] with permission from Elsevier).

When maternity care does not meet best practice standards and pregnant people receive substandard care, it is understood as another form of disrespectful, inequitable care. Women enter maternity care with an expectation that the facility and their provider base their care and recommendations on current best practices.^6^ When interventions are executed poorly, best‐practice interventions are not offered, and when interventions are performed without women providing meaningful consent (non‐consented care), these are all forms of substandard care.^12^ This includes when insufficient information has been provided to the patient to enable her to make an informed decision.^12^ Figure 1 illustrates practices recognized as substandard and disrespectful maternity care as described in a recent statement.^12^

**TABLE 2:** Forms and contexts of disrespect and abuse of newborns.

Category	Description	Potential impact	References
Physical mistreatment	Slapping, rough handling, holding upside down or by limbs	Pain, trauma, compromised safety	[15, 16, 17]
Non‐consented or unauthorized care	Diagnostic or therapeutic interventions without parental consent	Ethical violations, mistrust, legal repercussions	[16, 17]
Unnecessary separation from caregivers	Separation from mothers without medical indication	Impaired bonding, disrupted breastfeeding	[15, 17]
Discriminatory practices	Based on sex, ethnicity, age of mother, socioeconomic status	Inequity in care, stigmatization	[15, 16]
Neglect and inadequate medical attention	Failure to provide timely and appropriate care, especially for preterm/small‐for‐gestational‐age infants	Increased morbidity and mortality	[15, 16, 17]
Systemic failures	Early discharge, lack of staff/equipment, detainment due to inability to pay	Structural barriers, rights violations	[16]

## 4 INCIDENCE OF DISRESPECTFUL CARE AND WORLDWIDE STATUS

The incidence of disrespectful care has been reported as between 11% and 33% in large‐scale studies in Australia, Norway, and Mexico,^18^, ^19^, ^20^ and a recent meta‐analysis found a global prevalence of 59% with data from 15 countries.^21^ In the meta‐analysis, the most common form of disrespectful care was non‐consented care, affecting 37% of women.^21^ In Mexico, a national survey (*n* = 24 126) found that 23.6% of women experienced “obstetric violence,” with an additional 17.1% subjected to non‐consensual care.^20^ Further, a study in Tanzania involving nursing and midwifery staff found that 96.1% of participants (*n* = 422) reported acts of disrespect towards patients.^22^, ^23^ A study in Ethiopia (*n* = 2493) reported a 49% pooled prevalence of disrespect during childbirth, with instances of physical abuse, non‐confidential care, abandonment, and detention.^24^

### 4.1 The impact of disrespect on women and newborns

Disrespectful practices in maternity care are not only violations of women's fundamental rights as outlined by the Universal Declaration of Human Rights,^25^ but they also have direct and indirect repercussions on maternal and neonatal outcomes. Disrespectful care has been directly linked to increased incidence of postpartum depression, post‐traumatic stress disorder, and diminished maternal–infant bonding.^26^, ^27^, ^28^, ^29^ Women often avoid health facilities due to previous experiences of poor‐quality care, discrimination, and neglect.^30^ Practices such as enforced separation from family during labor and insistence on supine birthing positions are both disrespectful and contradict best‐practice recommendations. Women have expressed that a sense of personal achievement and retaining control are both essential for a positive experience of childbirth.^30^

## 5 PROVIDING RESPECTFUL CARE

In parallel with efforts to define and understand the ways disrespect manifests in maternity care, research has been undertaken to describe how healthcare providers might act with respect and compassion. Krausé et al. identify three core components of compassionate care provision by healthcare providers:

- Establish meaningful connection: Build trust, demonstrate strong interpersonal skills, and treat women with dignity.
- Treating women as individuals is also described as person‐centered care: being receptive to individual experiences, values and needs, and supporting and respecting women's choices.
- Perform actions with care: Provide continuous information and emotional support.^31^

### 5.1 Person‐centered care

The WHO recognizes person‐centered care as an essential element for improving health outcomes. This approach respects individuals' goals, values, and preferences, empowering them to participate actively in healthcare decisions.^32^ To provide person‐centered care, it is vital that healthcare providers effectively communicate relevant information about the choices women have in the context of their values and with the goal of improving their ability to make autonomous decisions. To enable women to have a safe and positive experience, healthcare providers should orient women during antenatal care to identify their options should an unexpected, high‐risk situation occur during labor and birth. These discussions should be documented. Women should be prepared to make rapid decisions with their healthcare providers if required when there is a threat to maternal or perinatal health. Providers should be prepared to work with women whose decisions for themselves and their babies might differ from those the providers would prefer. When emergency interventions are performed to avoid maternal or perinatal morbidity or mortality, this should only be with the consent of the woman. If the woman is unable to give consent, providers should ensure they return to debrief about the actions that were taken and discuss any concerns the family might have about the care that they received. Conversations during prenatal care help women set reasonable expectations and prepare them to make choices that align with evidence‐based and feasible non‐harmful practices.^12^

### 5.2 Trauma‐informed care

Pregnancy and childbirth might cause women to feel vulnerable, placing them at risk of a traumatic experience. This risk is even more pronounced for women who have experienced sexual assault. By way of example, it is estimated that 20% of women in the UK and the USA have experienced sexual assault.^33^, ^34^ Survived sexual assault have reported a need to retain decision‐making control during childbirth to maintain their feelings of safety.^33^

National and international academic societies have developed policies and practice recommendations to address sexual trauma and traumatic birth experiences. The American College of Obstetricians and Gynecologists promotes the universal use of a trauma‐informed approach because not all women will disclose their history; the UK's National Health Service likewise advises assuming any woman in perinatal care has experienced trauma or mistreatment.^35^, ^36^ Australia's 2023 National Perinatal Mental Health Guideline contains a review of guidelines that address traumatic births.^37^ The Society of Obstetricians and Gynecologists of Canada (SOGC) has created an online course for healthcare providers on “Trauma and violence‐informed care”.^38^ The Norwegian directorate recommends routine screening and referral for women who have experienced abuse or violence.^39^ Ongoing education for providers to build competencies for trauma‐informed care is recommended to promote trust and prevent negative birth experiences.

### 5.3 Culturally competent care

Culturally competent care involves tailoring health care to meet patients' social, cultural, and linguistic needs.^40^ Recommendations to improve cultural competency in maternity care include recruiting diverse staff members reflective of the patient population, routine training and assessment of skills for cultural competency, availability of interpreters and translated media, and accommodations for cultural preferences to the extent possible.^40^ Cultural safety expands on cultural competency to recognize the systemic inequities that affect patient care, leading to ineffective care and potentially harmful dynamics. It asks providers to reflect on their culture and historical colonial factors that create power imbalances in the provider–patient relationship.^41^, ^42^ Cultural safety requires introspection and extrospection at the individual and institutional levels. It requires providers to support service users to help define and create a culturally safe space for their care.^42^

### 5.4 Respectful care requires informed decision‐making

To provide evidence‐based care while offering women choices, the WHO guidelines for intrapartum care (2018) provide specific recommendations for how providers can protect women's autonomy.^43^ The care provided should be based on her choice once the provider has given her the relevant, accurate, and understandable information on risks and benefits.^43^ This includes decisions on, for example, labor positions, elective induction of labor, or use of epidural analgesia.^43^ Shared decision‐making is recommended, where options are discussed based on evidence and women's values, beliefs, and preferences. Providers must avoid offering interventions unsupported by scientific evidence of benefit (e.g., episiotomy).^6^ The woman should always have the final say where she has capacity. Being in labor or under local anesthesia (epidural) does not incapacitate women from being decision‐makers in their own health and treatments.

Routine interventions without consent might have unexpected negative impacts. A study in Kenya (*n* = 1014) found that women asked to consent to procedures and exams for their newborn were 27% more likely to attend postpartum visits and 33% more likely to breastfeed exclusively after 10 weeks.^44^ Obtaining consent from women is more than a formality; it builds trust with the care team, resulting in more effective and cohesive care.

## 6 DEFINING DISRESPECT AND ABUSE OF NEWBORNS

While increasing attention has been directed towards improving respectful maternity care, significantly less focus has been given to the quality and respectfulness of care for newborns in the postnatal period.^6^, ^15^, ^16^, ^17^ A growing body of evidence reveals that mistreatment of newborns occurs not only in the immediate moments after birth but can also persist through the early postnatal phase.^15^, ^16^, ^17^ This mistreatment encompasses a spectrum of harmful behaviors and systemic failures, including physical abuse (e.g., slapping, rough handling, and holding newborns upside down or by their limbs), neglect, unwarranted separation from caregivers, delays or denials of appropriate medical care, and provision of diagnostic or therapeutic interventions without informed consent (Table 2).^17^ Such violations are further compounded by discriminatory practices, often targeting neonates born to adolescent mothers, marginalized ethnic communities, or those perceived as socioeconomically disadvantaged. Particularly concerning is the vulnerability of preterm and small‐for‐gestational‐age infants, who face elevated risks of neglect and inadequate care.

From a health systems perspective, these occurrences reflect deeper institutional and infrastructural shortcomings. Disrespect and abuse of newborns often emerge in contexts of under‐resourced healthcare settings where high patient loads, insufficiently trained staff, and lack of essential supplies and equipment undermine safe and compassionate care. Further, institutional policies that overlook the rights and needs of neonates exacerbate the problem. Practices such as premature discharge without appropriate evaluation, non‐consented referrals, and even detainment of mothers and infants for inability to pay, expose fundamental equity and accountability deficits in the system.^16^ Advocacy for neonatal respect must be embedded within broader frameworks of respectful maternity care to ensure continuity and consistency across the perinatal spectrum.^15^, ^17^ Looking ahead, establishing clear, measurable standards for respectful newborn care is paramount. Future research must define operational indicators, while policy frameworks should integrate training, resource allocation, and legal protections to uphold neonatal rights. A systemic shift is required—one that not only recognizes but actively safeguards the dignity of all newborns, regardless of their birth circumstances. Creating such systems is not just a clinical necessity but a moral imperative.

## 7 THE INTERNATIONAL CHILDBIRTH INITIATIVE: A PLATFORM FOR RESPECTFUL CARE AND ACCOUNTABILITY WITHIN HEALTH FACILITIES

FIGO has long recognized that every woman has the right to dignity, respect, and skilled care during pregnancy and childbirth. Respectful care should be available for women regardless of whether they have a low‐ or high‐risk pregnancy and whether they live in high‐, middle‐, or low‐income countries. In 2018, FIGO partnered with other global leaders (International MotherBaby Childbirth Organization [IMBCO], International Confederation of Midwives, International Pediatric Association, and White Ribbon Alliance) to create the International Childbirth Initiative (ICI). The Initiative recognizes facilities that apply to implement the 12 Steps to Safe and Respectful MotherBaby‐Family Maternity Care through a process intended to develop local leadership and create sustainable improvement of care.^1^, ^2^ All facilities are encouraged to apply for recognition as part of the ICI network to access tools and support for respectful care (icichildbirth.org/application) (Box 2).

BOX 2 **Respectful care for newborns and stillborn under the International Childbirth Initiative (ICI) 12 Step Framework^45^**

**Table:** 

International Childbirth Initiative Framework	Specific needs of newborns or stillborn
Step 1: Respect and compassion for mother and newborn	Only medically necessary separation of mother and newborn (with consent of mother) Newborn handled in gentle and safe ways. All procedures and referral are consented by the caregiver. Stillborn infants are given respect and choices offered to the family about how to grieve or memorialize infant, including supported opportunities to engage in parenting according to their needs and preferences, such as naming, seeing, holding and meeting the newborn.
Step 2: Provide free or affordable and accessible and non‐discriminatory care	Services provided for women and newborns with disabilities
Step 6: Evidence based practice	Provision of clean, warm, safe environments for delivery and newborn care. Staff confident in providing essential newborn care
Step 7: Avoid unnecessary procedures	Avoidance of unnecessary, painful procedures for newborn.
Step 8: Preventative care	Prompt removal of soiled wrapper or diaper and cleaning of urine and feces.
Step 9: Provide emergency care	Staff trained to handle critically ill newborns. Equipment is available, clean and ready for low birth weight or preterm infants and other potentially critical newborns
Step 11: Provide a continuum of collaborative care	Staff trained in compassionate care for bereaved families, and counseling services available Separate postpartum area for parents of stillborn infants available
Step 12: Skin‐to‐skin and breastfeeding support	Supportive breastfeeding counseling and demonstrations

## 8 INDICATORS FOR MONITORING RESPECTFUL PRACTICE

Many gaps remain to be closed to ensure that national and individual health facility policies follow evidence‐based practice and support women's rights in childbirth. Policies and accountability measures promoting respectful care are effective in reducing the number of women and newborns who experience disrespect.^45^ We understand that meaningful change requires the measurement of disrespectful care and recommend routine collection of measurable indicators. To this end, FIGO has supported ICI to develop questionnaires and tools, which are currently available in 25 languages. These tools support the collection of data through a review of facility policies, an annual review of statistics, and by surveying representative samples of women and their chosen companions at least twice per year.

The following key indicators are drawn from the ICI tools, which can be accessed following an application or on request (icichildbirth.org/application). These indicators align with the WHO's standards for improving quality of maternal and newborn care and facilitate standardization across settings.

Indicators evaluated through policy assessment

- A formal system exists for women to file a complaint; this system is accessible, and women are routinely informed of how to file a complaint.

Indicators evaluated twice per year or annually

- Documentation that women received thorough counseling and subsequently either accepted or declined medical procedures that were offered
- Number of complaints submitted through formal complaint system or through women's questionnaire responses.

Health facility teams should routinely review data from women's and companions' questionnaires for the following indicators. This data should also be disseminated to staff to improve understanding of performance and expectations.

Women and their companions report whether:

- Privacy was protected during labor/delivery
- They were treated the same as other women.
- Providers introduced themselves and explained their role
- Providers used language that was understandable
- Women were able to ask questions or voice concerns
- Providers cared about women's values
- Providers encouraged women to have positive attitudes towards their body's capacity to give birth without medical intervention
- They were not subjected to physical mistreatment
- They were not subjected to verbal or emotional mistreatment
- They were allowed to have companions during labor/delivery
- Neither woman nor newborn received an intervention without her consent
- The newborn remained with the mother/family.

### 8.1 Staffing conditions

In addition to the previously mentioned factors, working conditions are an important consideration. Respectful care will not be achieved when hospital staff feel undervalued or treated disrespectfully by their superiors, the facility, or the health system.^46^ Low wages, excessive criticism, rigid working conditions, excessive shifting to other departments, and uncomfortable working environments all lower staff morale and performance.^46^ To create an environment for respectful care, healthcare providers must be treated well, and senior staff need to model and uphold respectful treatment at all levels.

### 8.2 Additional resources for changing practice and policy

Several training modules are in use or under development to support healthcare providers in providing respectful care. Caring for Providers to Improve Patient Experience (CPIPE) is an intervention to improve patient‐centered care with training modules to reduce provider stress, burnout, and bias with the understanding that these are contributors to disrespect.^47^ Evaluations of the impact on Kenyan health facilities found improvements in provider–provider and provider–patient relationships and reduced burnout.^47^ Project ARIISE was developed for use in American medical schools to teach skills for empathy and compassion.^48^ The International Confederation of Midwives developed the RESPECT Toolkit, an online resource available in English, Spanish, and French.^49^ Integrating these and similar resources into pre‐service curricula and in‐service training should be prioritized to give all healthcare providers the necessary tools to achieve higher standards of care.

Addressing the broad spectrum of challenges in maternity care demands systematic changes beyond the current focus on reducing financial burdens to families seeking respectful and high‐quality maternal and newborn care. An integrated approach involving the inclusion of midwives alongside obstetricians can significantly enhance care delivery. The WHO has provided guidance on financing healthcare systems to lessen the financial strain on families who struggle to afford necessary services.^50^ This restructuring is vital to avoid the detention of women and newborns in hospitals, reflecting a severe underfunding of hospitals, particularly in the public sector. Removing these financial burdens is important to reducing discrimination against patients and the inequitable care of those perceived as “unable to pay.” FIGO strongly recommends that antenatal, maternity, and postpartum care must be free for all. This approach reduces the financial barriers and helps curtail the mistreatment and discrimination against those perceived as unable to pay.

## 9 CONCLUSION

Ensuring respectful and compassionate care not only enhances the well‐being of mothers and babies, preventing potentially lifelong physical and mental trauma, but also fosters trust and confidence in healthcare systems. FIGO recommends an integrated team‐based approach to childbirth, emphasizing respectful care through evidence‐based treatments, avoiding harmful practices, protecting from violence, being aware that many women have already been traumatized, avoiding re‐traumatization, and ensuring culturally safe, family‐centered care and shared decision‐making that enables women to retain their autonomy throughout pregnancy, labor, and childbirth. By following these strategies, healthcare providers can empower women, respect their rights and preferences, and improve maternal and newborn outcomes.

## AUTHOR CONTRIBUTIONS

Conception: all authors. Research: HB, JM, MST. Writing: HB, JM, FP, MST, SM. Editing: HB, BJ, AL, SM, JM, DPB, MS, MST. Review: all authors.

## FUNDING INFORMATION

No funding was used in the preparation of the manuscript.

## CONFLICT OF INTEREST STATEMENT

The authors declare that they have no known competing financial interests or personal relationships that could have appeared to influence the work reported in this paper.

## Supporting information


Data S1:

## Data Availability

Data sharing is not applicable to this article as no new data were created or analyzed in this study.
