# Supplementary figures and images for: FIGO statement on respectful care: Addressing disrespectful maternity care
Source: Int J Gynaecol Obstet. 2025 Sep 30;171(3):983–92. doi: 10.1002/ijgo.70513 (PMC12640169; doi:10.1002/ijgo.70513)

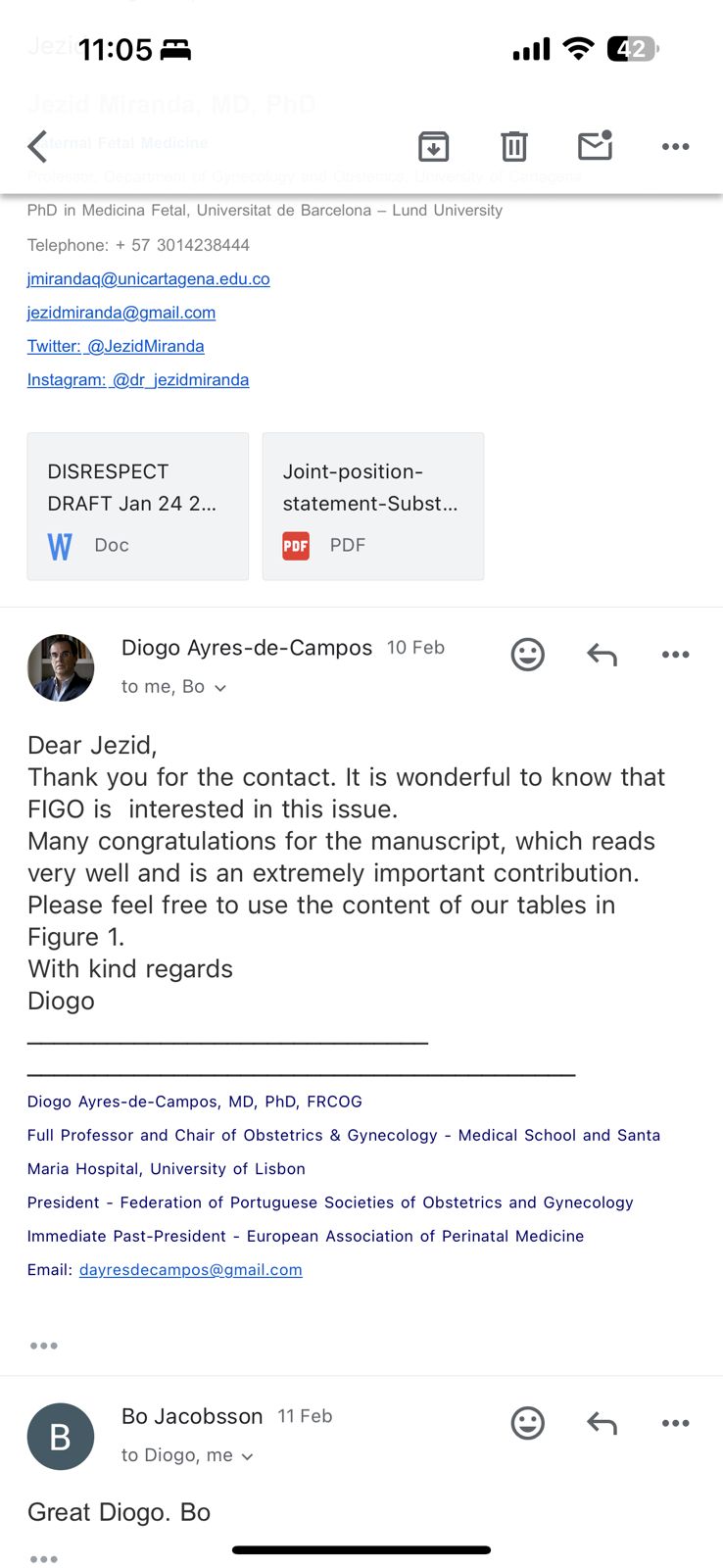

Supplement: Supplementary file 1 — Data S1: [file IJGO-171-983-s001.jpeg]
